# Selection of DNA aptamers specific for live *Pseudomonas aeruginosa*

**DOI:** 10.1371/journal.pone.0185385

**Published:** 2017-09-22

**Authors:** Jennifer Soundy, Darren Day

**Affiliations:** School of Biological Sciences, Victoria University of Wellington, Wellington, New Zealand; Laurentian, CANADA

## Abstract

*Pseudomonas aeruginosa* is an opportunistic pathogen that causes significant morbidity and mortality in immunocompromised patients, particular cystic fibrosis sufferers, burns victims, diabetics and neonates. It thrives in moist places where it forms biofilms that are exceedingly difficult to eradicate on hospital surfaces, in water supplies and implanted biomaterials. Using a live cell SELEX approach we selected DNA aptamers to *P*. *aeruginosa* grown as biofilms in microfluidic cells. From a pool of aptamer candidates showing tight binding a stem-loop structure was identified as being important for binding. Enhanced binding and increased specificity was achieved by truncating structures and generating chimeric aptamers from the pool of top candidates. The top candidates have low nanomolar binding constants and high discrimination for *P*. *aeruginosa* over other Gram-negative bacteria. The aptamers bind both planktonic grown and biofilm grown cells. They do not have intrinsic bacteriostatic or bactericidal activity, but are ideal candidates for modification for use as aptamer-drug conjugates and in biosensors.

## Introduction

*Pseudomonas aeruginosa* is a clinically important, opportunistic, gram-negative bacterium that is responsible for a wide variety of severe hospital-acquired infections. It largely effects immune-compromised patients, particularly those with cystic fibrosis, as well as causing infections in burn wounds, and forming biofilms on implanted devices such as urinary catheters and heart stents [1]. *P*. *aeruginosa* is intrinsically resistant to many anti-microbials due to the low permeability of its outer membrane, and upregulation of multidrug efflux pump systems [2]. Resistance is further accentuated by the formation of biofilms composed of a matrix of excreted exopolysaccharides, proteins and DNA that helps protect the bacteria inside from antibiotic action [3]. Bacteria within the biofilm also exhibit adaptive changes in gene expression and a shift to a metabolically less active state making the infections harder to clear, which in part accounts for the recalcitrance of many *P*. *aeruginosa* infections to antibiotic therapy.

Aptamers are short single stranded DNA or RNA oligonucleotides that can be selected to a wide variety of targets, but their potential for use as therapeutics targeting pathogenic bacteria has not been well explored. Aptamers are selected using a SELEX (Systematic Evolution of Ligands by Exponential Enrichment) methodology [4], that can be modified to select aptamers to living whole bacteria, viruses or mammalian cells [5–7]. Aptamers that have bacteriostatic activity against *Salmonella* and inhibitory properties against *Mycobacterium* have been reported [8, 9]. Aptamers are relatively non-immunogenic and non-toxic due to their small size, and can be rapidly chemically synthesised and modified, making them an interesting candidate for new drug development. They form stable secondary structures which allows them to bind to their target with high affinity and specificity, potentially inducing a therapeutic effect. Currently no aptamers have been developed to live *P*. *aeruginosa*, only to fixed bacteria [10].

In this study we describe the selection and characterisation of DNA aptamers selected to live biofilm derived *P*. *aeruginosa* cells that bind with a high affinity and specificity to *P*. *aeruginosa* but not to a selection of other gram-negative bacteria.

## Materials and methods

### Bacterial culture

The bacterial strain used for SELEX selection was *P*. *aeruginosa* 692 (PA692) (ATCC 14502) obtained from New Zealand Culture Collection (Porirua, NZ). Other bacterial strains used were: *P*.*aeruginosa* PAO1 (ATCC 15692), *Salmonella enterica serovar Typhi* (ATCC 19430), *Klebsiella pneumoniae* (ATCC 13883), *Enterobacter cloacae* (ATCC 13047), *Listeria innocua* (ATCC 33090) and *Escherichia coli DH5α*. Additional *P*. *aeruginosa* strains used in binding studies were clinical isolates PA1024 (catheter, urine), PA1205 (left thigh), PA1323 (cystic fibrosis sputum), PA1079 (tracheal aspirate), and PA1236 (blood) obtained from Environmental Science and Research Culture Collection (Porirua, NZ). All bacterial strains were cultured in standard Luria Broth (LB) medium at 37°C for 16 hours with aeration. After growth, the cells were diluted 1:100 in fresh LB and grown for 4 hours to allow them to reach exponential phase.

### Biofilm growth

The selection strain was grown as a biofilm in a microfluidic flow cell with an 800 μm channel depth (Ibidi, Germany). The flow cell was inoculated with 200 μL of exponentially growing culture diluted to an OD_600_ = 0.1. Cells were left to adhere for 2 hours prior to commencing flow of growth media (0.5x LB) at a flow rate of 1 mL/hr driven by a syringe pump (KD Scientific). Cultures were grown for 42 hours at ambient temperature (22°C) prior to washing loosely adhered planktonic cells away by increasing the flow rate to 5 mL/hr for 1 hour. The biofilm was collected by fluidic agitation and suspended in 1 mL 0.85% NaCl. Biofilm cells were washed three times in 0.85% NaCl with vortexing to dissociate and remove excess alginate and exopolysaccharides characteristic of biofilms. Cells were then washed by centrifugation and re-suspended in wash buffer consisting of phosphate buffered saline (PBS) containing 0.05% Tween 20 prior to washing with SELEX binding buffer (wash buffer, containing 1% BSA). The cells were re-suspended in binding buffer to a density of 10^6^ CFU/mL in preparation for SELEX selection.

### Whole-cell SELEX

Aptamers were selected against live *P*. *aeruginosa* cells grown as a biofilm using a modified whole-bacterium SELEX protocol [11]. The random ssDNA library (Integrated DNA technologies, USA) consisted of a random 45 nucleotide site flanked by two constant primer regions, ATGAGAGCGTCGGTGTGGTA-N_45_-TACTTCCGCACCCTCCTACA. Before cell-SELEX the ssDNA library was denatured by heating at 95°C for 5 min then snap cooled on ice for 10 min. For the first round of selection 1 nmol at 1 μM of ssDNA was incubated with bacteria in 1 mL binding buffer for 30 min at room temperature (22°C) with gentle agitation. Aptamer bound cells were recovered by centrifugation (3,500xg, 10 min) and washed twice with 1 mL wash buffer to remove unbound oligonucleotides. Washing time and volumes were increased in subsequent rounds to increase the stringency of selection. Bound oligonucleotides were recovered by re-suspending cells in 100 μL of TE buffer (10 mM Tris-HCl pH 8.0 containing 1 mM EDTA) and heating to 95°C for 5 min then centrifugation at 14,000xg for 15 min. The supernatant that contained the eluted candidate sequences was kept and used for PCR amplification to generate the next library.

Library amplification was monitored by real time PCR using a CFX-Connect Real Time PCR Detection System (Biorad, USA) with SYBR green chemistry to determine the optimal number of amplification cycles to prevent the formation of non-specific amplification products [11] (S1 Fig). Amplification was performed using SensiMix SYBR & Fluorescein mix (Bioline, UK), with 500 nM forward primer (5’- ATGAGAGCGTCGGTGTGGTA-3’) and 500 nM reverse primer (5’- TGTAGGAGGGTGCGGAAGTA-3’) (IDT, USA) in a total volume of 500 μL. A thermal cycle consisting of an initial enzyme activation step of 95°C for 10 min, then 95°C for 10 sec and 65°C for 15 sec for the optimised number of cycles was used for amplification and the quality of the PCR product was confirmed by agarose gel electrophoresis. The single stranded DNA library was regenerated by asymmetric PCR [12]. The asymmetric PCR was performed using 50 μL of the dsDNA PCR product as template in a final volume of 500 μL. Amplification was achieved using KAPA2G HotStart DNA polymerase (10 U) (KAPABiosystems, USA) with the supplied KAPA2G buffer A, and contained 0.2 mM dNTPS, 1 μM FAM labelled forward primer (5’-FAM-ATGAGAGCGTCGGTGTGGTA-3’) and 50 nM reverse primer. The PCR was run for 20 cycles using the same cycling conditions. The ssDNA PCR product was desalted using an Illustra NAP-5 column (GE Healthcare, UK) in to wash buffer and stored at -20°C until the next round of selection. ssDNA concentration was determined by 3% agarose electrophoresis and densitometry analysis against known concentration standards using ImageJ [13].

### Cloning and sequencing of aptamers

After the 7^th^ round of selection the evolved aptamer pool was PCR amplified using unmodified primers and purified using the QIAEX II Gel Extraction Kit (Qiagen, Germany). Cloning was performed using the TOPO-TA cloning kit (Thermo Fisher Scientific, USA) following the manufacturer’s instructions. White colonies were picked and grown individually overnight at 37°C, before being analysed for the correct sized insert by PCR using the aptamer specific primers. Plasmids from clones containing the desired insert were isolated using the GeneJET plasmid miniprep kit (Thermo Fisher Scientific, USA) following the supplied protocol and were sequenced by Macrogen Inc. (Korea) using the M13R-pUC primer. Aptamer candidates were synthesised (IDT, USA).

### Flow cytometry

Flow cytometry was used to assess binding of the aptamer libraries and the aptamer candidates. Cells were grown in LB to an OD_600_ = 0.1, washed once in flow buffer (1x PBS containing 0.1% BSA), then re-suspended in 200 μL of flow buffer. A BD FACSCanto II flow cytometer (BD Biosciences, USA) was used to identify bacterial cells based upon forward and side scatter profiles. Aptamer binding was performed in a volume of 300 μL of flow buffer containing 10 μL of cell suspension and the desired concentration of library or aptamer. The samples were incubated at room temperature for 15 min with aptamer, collected by centrifugation (13,000xg, 2 min) then re-suspended in 300 μL PBS for flow analysis. At least 10,000 events in the gated bacterial population were collected and data analysed using Flowing Software 2.5.1 (Turku Centre for Biotechnology, Finland).

The dissociation constants were determined by the same method, incubating bacterial cells with aptamer candidate concentrations in the range of 1 nM to 250 nM in triplicate experiments. Specificity of binding was confirmed by performing experiments in parallel in which *E*.*coli* replaced *P*. *aeruginosa* as the target bacteria. Non-specific binding was subtracted from total binding and dissociation constants calculated using GraphPad Prism 5 with a one site specific binding model.

### Metabolic activity

To determine whether aptamer binding induced changes in metabolic activity incorporation of 5-cyano-2,3-ditolyl tetrazolium chloride (CTC) was used. CTC is oxidised to an insoluble fluorescent precipitate by metabolically active cells [14]. Cells were incubated with aptamer for 15 min then 5 mM CTC was added and incubated for a further 15 min before being analysed by flow cytometry. Changes in cell permeability were determined by staining with propidium iodide (2 μg/mL, 1 min) and flow cytometry analysis.

### Aptamer effects on bacterial growth

Bacterial growth was monitored by measuring the increase in turbidity at 600 nm as a function of time (growth assays) in 96-well plates using an Enspire 2300 Multilabel plate reader (Perkin Elmer, USA). Exponentially growing bacteria were washed and re-suspended in LB and approximately 2x10^5^ CFU added to each well with 1 μM of unlabelled aptamer in a total volume of 200 μL. Wells with LB only, and untreated bacteria were used as controls for contamination and normal growth respectively. Each condition was performed in triplicate and OD_600_ readings were taken every 30 minutes for 16 hours at 37°C.

## Results

### Aptamer selection

**Fig 1:**
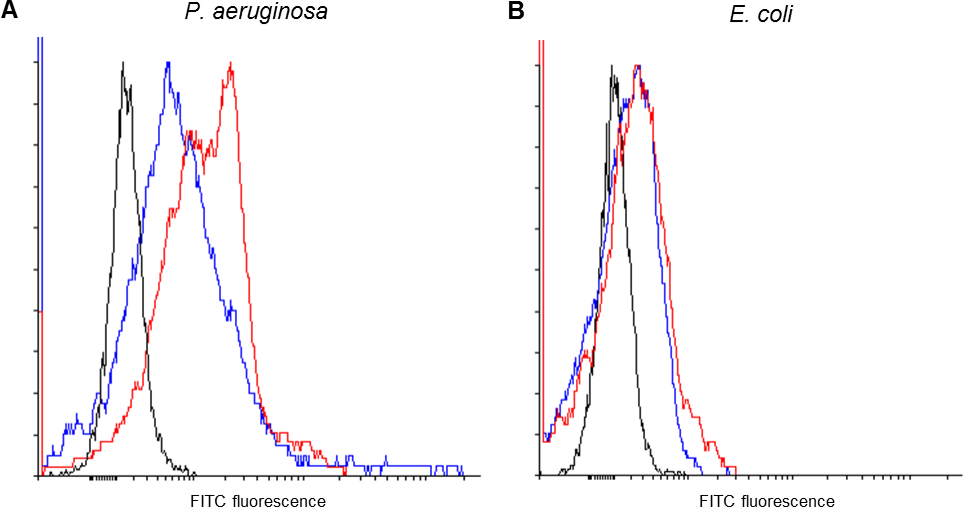
The seventh SELEX round shows enhanced binding to *P*. *aeruginosa*. Binding of the seventh SELEX round (red) was assessed relative to the starting library (blue) to *P*. *aeruginosa* (A) and *E*. *coli* (B). The fluorescence of unstained cells is indicated in black. The seventh SELEX round shows increased binding to *P*. *aeruginosa* (right shift in the histogram) relative to the starting library, but both the starting library and the seventh SELEX round bind equally poorly to *E*. *coli*.

Aptamers were selected to *P*. *aeruginosa* bacteria grown as a biofilm with whole-cell SELEX being undertaken for seven cycles. Flow cytometric analysis of fluorescently labelled aptamer pools was carried out to assess the binding affinity of each library as selection progressed. After seven rounds of selection the library (L7) showed enhanced binding relative to the initial starting library (L0) so further selection rounds were not undertaken as it was unclear whether further enrichment would be achieved relative to the potential loss of tight binders (S2 Fig). Fig 1A shows that both the L0 (blue) and L7 (red) libraries bind to *P*. *aruginosa*, as the median fluorescence intensity is greater than the unstained cells (black), but the L7 library had enhanced binding relative to the non-specific staining of the L0 library. This increased binding was not seen for *E*. *coli* cells (panel B) as both the L0 and L7 bound approximately equally. The seventh library was thus cloned in to *E*. *coli* and sequenced to identify potential aptamer sequences.

### Screening of aptamer candidates and binding affinity determination

Single-stranded aptamer candidates were prepared from the plasmids by asymmetric PCR using a fluorescently labelled primer to allow analysis by flow cytometry, and changes in metabolic activity and membrane permeability were determined by staining with CTC and PI respectively. Enzymatic preparation of aptamer candidates rather than chemical synthesis was undertaken as an initial screen to reduce costs as only small amounts of each candidate was required. The aptamer candidates were ranked according to binding affinity (shift in median fluorescence intensity of stained cells). Samples were also tested for their ability to alter metabolic activity or membrane permeability as previous studies have identified some bacterial aptamers that have bacteriostatic activity and depolarise cells [8]. No significant changes in CTC incorporaton or PI staining were detected but differences in binding were determined. Four candidates, JN17, JN21, JN08 and JN27, were selected as they were tight binders. The top two candidates, JN08 and JN27 were chemically synthesised to allow further characterisation.

**Fig 2:**
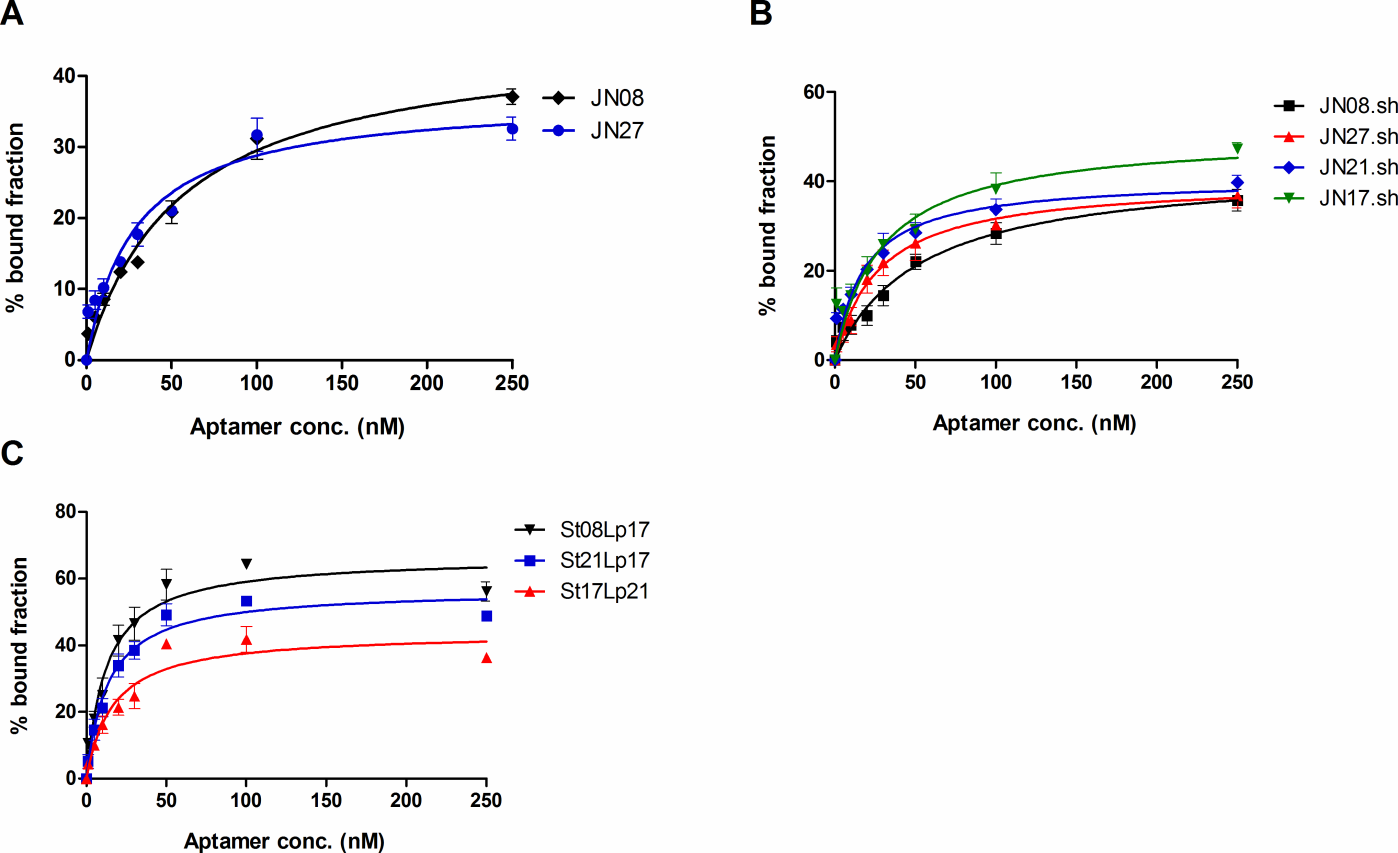
Dissociation curves of aptamer variants. Plots show mean ± SEM, n = 3 for each aptamer. A: original aptamers, JN08 and JN27, B: truncated aptamers, C: chimeric aptamers.

The secondary structures of the aptamers were predicted using mfold software [15] set for conditions used in binding assays (150 mM Na^+^ and 10 mM Mg^2+^, at 22°C). The secondary structure of JN27 indicated a single stem-loop as being the only significant stable structure suggesting this structural motif may be important for binding. To test whether this motif was important a truncated version comprising just the stem-loop (JN27.SH) was synthesised and it’s binding compared to the full length aptamer by incubating *P*. *aeruginosa* cells with differing concentrations and determining the median fluorescence of cells by flow cytometry (S3 Fig). Fig 2A shows the plots used for calculating the dissociation constants for JN08 and JN27. Fig 2B showed that the truncated versions that just contained the stem-loop structure identified by mfold also bound tightly (black and red curves). Having confirmed the stem-loops as being important, we reasoned that related stem loop motifs may be present in other aptamer candidates and that the structure may also be important for binding. To test this mfold was used to predict the secondary structures of the top binding candidates identified from the initial screen. Several of the tighter binding candidates had similar stem-loop motifs, so truncated versions were prepared and their binding evaluated. The truncated aptamer candidates JN17.SH, JN21.SH and JN08.SH all showed tight binding to *P*. *aeruginosa* (Fig 2B).

**Fig 3:**
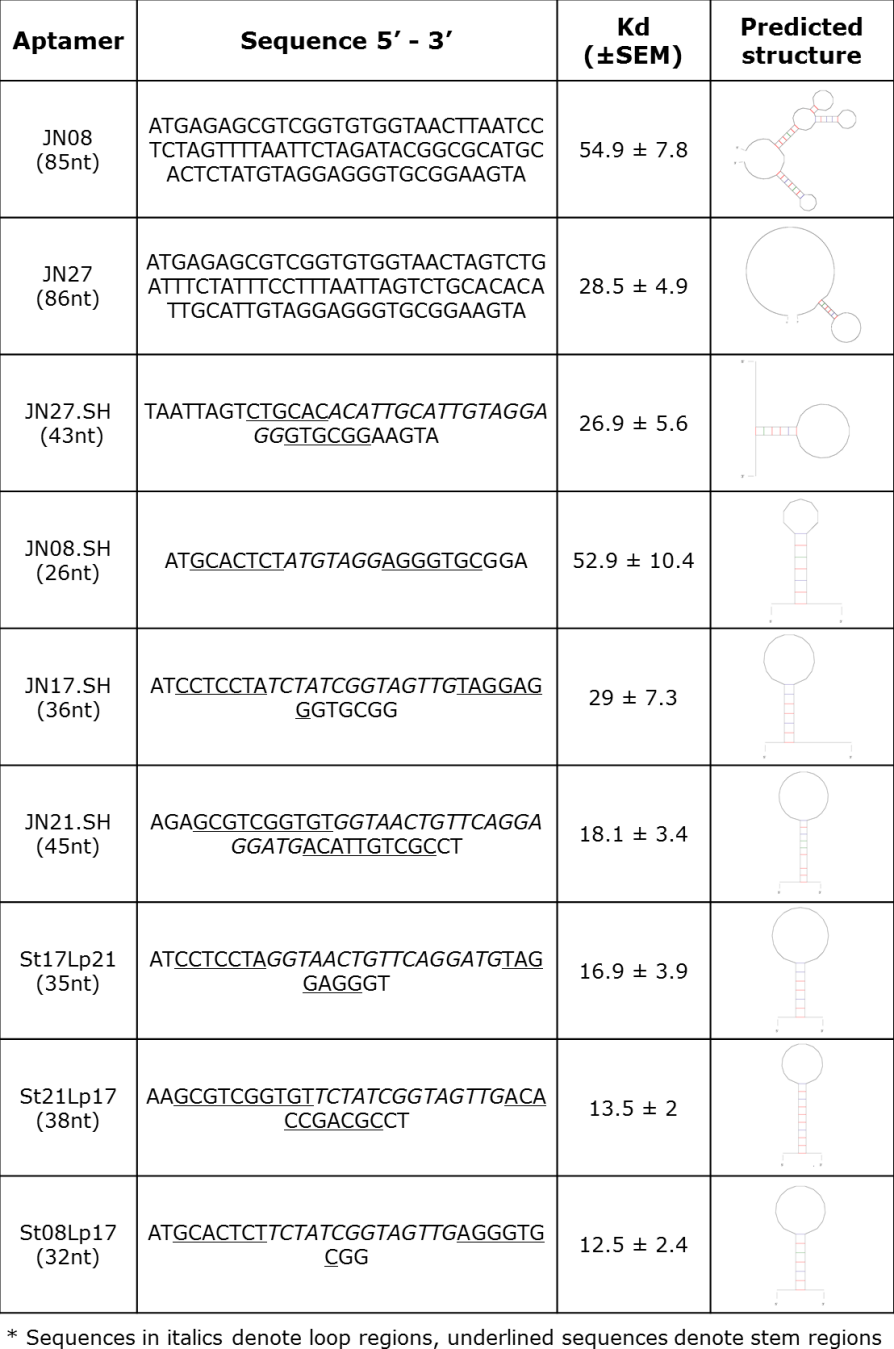
Aptamer sequences and their dissociation constants (Kd).

To further investigate the stem loop motifs, *in silico* mating was done to generate new stem loop combinations using a mix and match of stem (St) and loop (Lp) structures. Three new chimeric aptamers, St21Lp17, St17Lp21 and St08Lp17 showed enhanced binding (Fig 2C) with Kds in the low nanomolar range (10–55 nM). All aptamer sequences, secondary structures and respective dissociation constants are shown in Fig 3.

### Determination of species specificity

**Fig 4:**
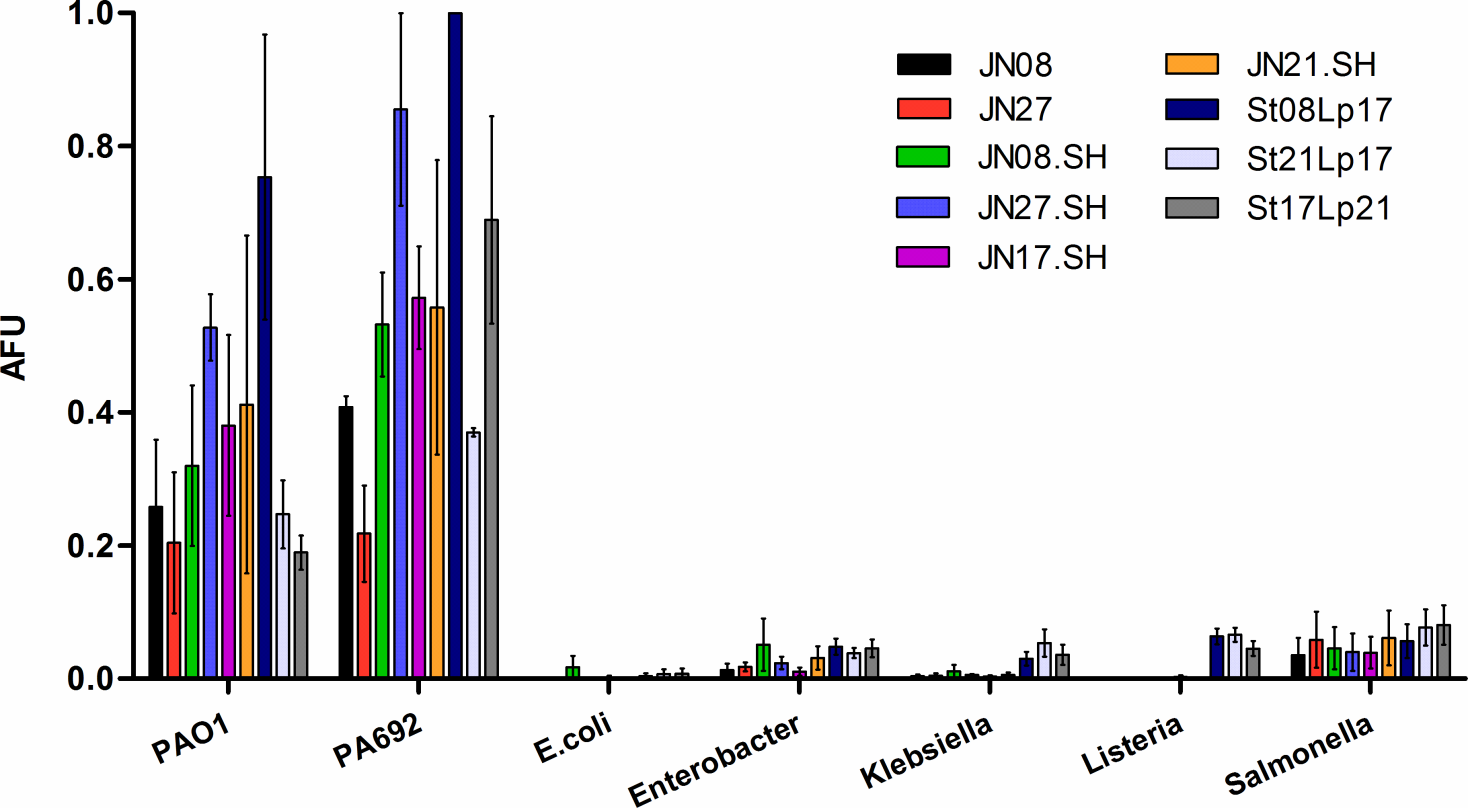
Specificity of aptamer binding to *P*. *aeruginosa* strains compared with other bacterial species. FAM-labelled aptamers (250 nM) were incubated with *P*. *aeruginosa* strains *(*PA692 and PAO1) or *S*. *enterica*, *K*. *pneumoniae*, *E*. *cloacae*, *L*. *innocua* and *E*. *coli*. Cells were washed, re-suspended in PBS and analysed by flow cytometry to measure the relative fluorescence intensity (arbitrary fluorescence units, AFU) due to bound aptamer (n = 3). All aptamer candidates preferentially bound the *P*. *aeruginosa* strains. A two-way ANOVA analysis showed a statistically significant interaction between the type of bacteria and different aptamer binding effects (F(48,126) = 2.42, p = <0.0001). The different bacterial species statistically significantly affects the results (F(6,126) = 98.91, p = <0.0001). Bonferroni post tests show aptamer binding intensities with PAO1 vs all other non-*pseudomonas* strains are statistically significantly different (P<0.05) for all aptamers except St21Lp17, St17Lp21, JN08 and JN27. Aptamer binding intensities between PA692 vs all other non-*pseudomonas* strains are statistically significantly different (P<0.05) for all aptamers except JN27. There is no statistical significance between non-*pseudomonas* strains.

To determine whether the aptamers were selective for *P*. *aeruginosa*, the identified aptamers were evaluated for binding to other Gram-negative bacterial species (*Salmonella enterica serovar Typhi*, *Klebsiella pneumoniae*, *Enterobacter cloacae*, *Escherichia coli DH5α)* and the Gram-positive bacteria *Listeria innocua*, in comparison with *P*. *aeruginosa* laboratory strains PA01 and PA692 (Fig 4). All aptamers were selective for *P*. *aeruginosa* with minimal cross-reactivity to the tested bacteria.

**Fig 5:**
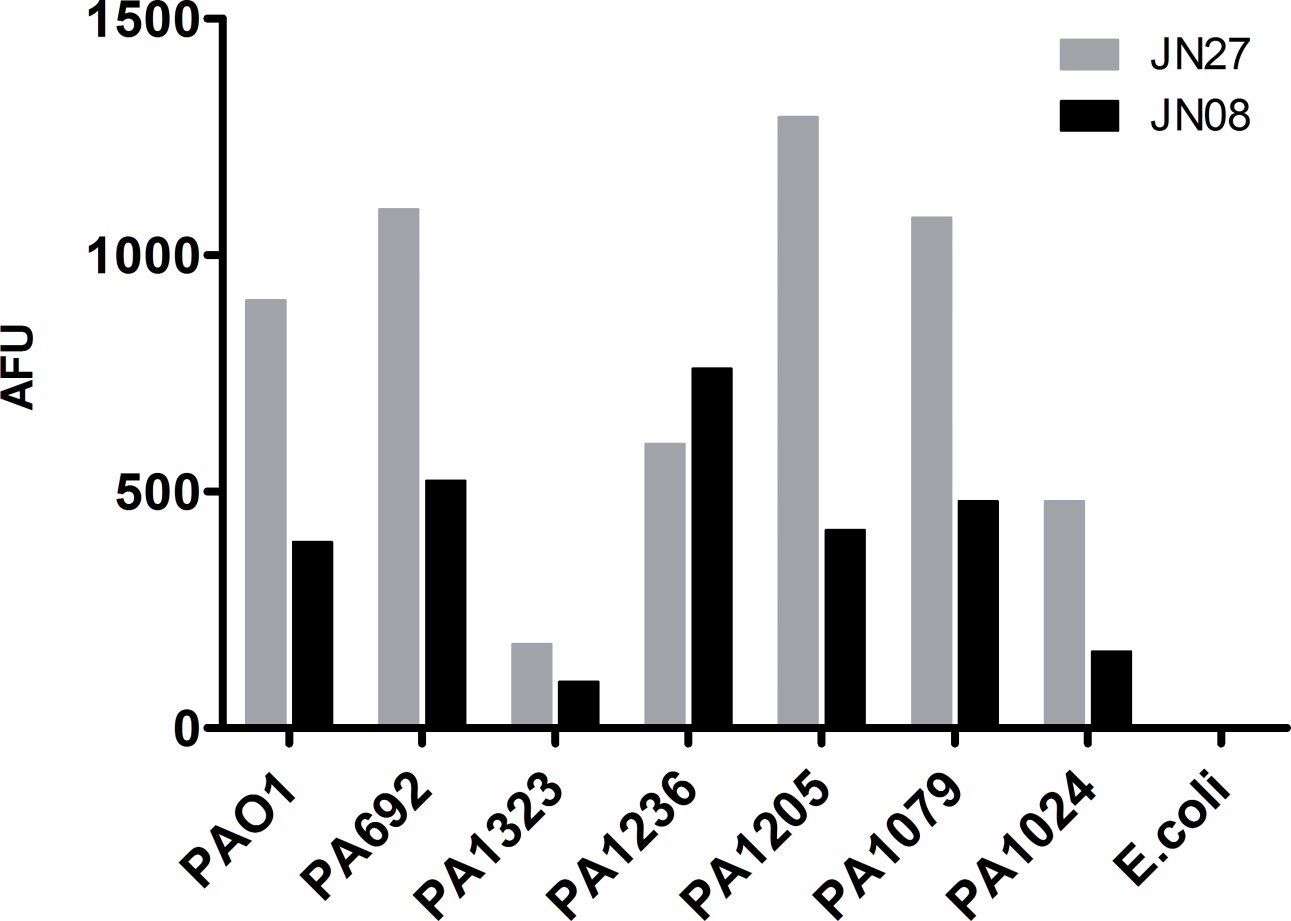
Aptamer binding to clinical isolates of *P*. *aeruginosa*. FAM-labelled aptamers (250 nM) were incubated with *P*. *aeruginosa* lab strains, clinical isolates, or *E*. *coli*. Cells were washed, re-suspended in PBS and analysed by flow cytometry to measure the relative fluorescence intensity (AFU) due to bound aptamer (n = 1).

To determine whether the aptamers were able to bind to clinical isolates of *P*. *aeruginosa*, five clinical isolates were tested alongside the two laboratory strains PA01 and PA692 as a positive control for binding, and *E*. *coli* as a negative control. Fig 5 shows that JN27 and JN08 were able to bind to the clinical isolates, albeit with differing efficiences. In general JN27 gave more intense staining than JN08, although neither aptamer recognised strain PA1323 as well as the others. In all cases staining was greater than that for *E*. *coli* which was neglible.

### Determination of intrinsic antibacterial effects

As some aptamers have been reported to have intrinsic antibacterial effects [8], each of the aptamers were tested for possible bacteriostatic or bactericidal effects using growth assays (S4 Fig), CTC staining, and increased PI uptake following a 15 min incubation with 1 μM aptamer. Our aptamers had no intrinsic bacteriostatic and/or bactericidal activity, even though they are highly specific for *P*. *aeruginosa*.

## Discussion

*P*. *aeruginosa* is an important bacterial pathogen that causes significant morbidity and mortality. It is now listed as the number two threat on the World Health Organisation list of anti-microbial resistant bacteria (2017) [16]. Current therapeutic options are limited due to *P*. *aeruginosa’s* intrinsic multi-drug resistance mechanisms and new therapeutics are urgently required. In this study we used a whole-cell SELEX procedure to select ssDNA aptamers against live, biofilm derived PA692 *P*. *aeruginosa* cells. While our aptamers were derived from cells grown as bioflms they bound live planktonically grown *P*. *aeruginosa*. The aptamers binding to live cells was confirmed by SYTO9/PI (live/dead) staining of planktonically grown cultures. In our hands the exponentially growing cultures used for testing contained more than 90% live cells based upon the PI staining profile during flow cytometry analysis and we isolated a number of aptamers that bind to these cells with high affinity and specificity, and evaluated the top candidates for antibacterial activity.

The cell-SELEX approach has been used to develop aptamers to both prokaryotic and eukaryotic cells, with much effort being directed toward developing tumour-specific aptamers. The cell-SELEX approach is particularly powerful as the epitopes that differentiate between tumour types, or in our case, bacterial species need not be known. The identification of the bound antigen is ultimately beneficial as it may allow rational modification of the aptamer sequence to tailor binding parameters, however, identification of the binding target is not a trivial undertaking, and was not investigated in this study.

Secondary structure analysis revealed a putative stem-loop motif that was common amongst the tighter binding candidates isolated from the final selection round. Truncation of the structures confirmed this stem-loop as being important for binding, and that truncation in most cases increased binding. Development and testing of chimeric aptamers with mixed stem and loop sequences from different aptamers corroborated our hypothesis that the stem loop structure was important as the chimeras showed tight, and for some, improved binding affinity. The ability of aptamers to show neutralising activity upon binding to cell surface receptors or ligands is well reported, but there are few reports describing antimicrobial activity caused by aptamer binding. While the aptamers we generated don’t have intrinsic bacteriostatic or bactericidal activity, their high affinity and specificity for *P*. *aeruginosa* makes them ideal candidates for use in biosensors or as the targeting moiety of conjugated therapeutics. The use of aptamers in biosensors is a rapidly growing field as aptamers are ideally suited for this purpose. The detection of *P*. *aeruginosa* in water supplies or moist environments such as in hospitals where it forms biofilms inside taps and on equipment is highly problematic, especially in neonatal wards where an opportunistic infection is usually fatal. We are currently evaluating our aptamers for use in biosensors to detect *P*.*aeruginosa* shed from biofilms.

Aptamers also have great potential for use as the targeting moiety for therapeutics and their use as such has been well reviewed [5, 17, 18]. Surprisingly, there are very few reports detailing aptamer targeting of antimicrobial compounds to bacteria. Aptamer targeting of antimicrobials offers great potential for increasing the therapeutic index of existing antimicrobials by allowing a greater effective concentration to be delivered to the microbes at the site of infection. This has the advantages of allowing the use of lower systemic doses to reduce patient toxicity, whilst also reducing the acquisition of resistance by achieving a higher concentration at the microbial cell. We believe they have strong potential for use in therapeutic applications and our group is currently pursuing this avenue of research.

To the best of our knowledge, we are the first to report DNA aptamers derived to live *P*. *aeruginosa* cells.

## Supporting information

S1 Fig qPCR monitoring of aptamer library amplification. Aptamer PCR amplification can be monitored by real time PCR using SYBR green interchelation. Agarose gel electrophoresis was performed on samples after 7, 9, 11, 13 and 15 cycles of amplification (lanes 1 to 5 respectively).
(TIF)

S2 Fig Relative binding affinity of aptamer libraries during SELEX progression. The relative binding of the aptamer libraries was determined by incubating 3x10^10^ biofilm bacterial cells collected from the flow cell with 100 nM of library in binding buffer for 30 min at room temperature. Samples were washed 3x, eluted and quantified by qPCR using the PCR protocol described in SELEX methods. The plot shows the change in the threshold cycle for amplification (Cq) relative to the initial starting library.
(TIF)

S3 Fig Comparison of binding affinity of JN27 and JN27.SH. Increasing concentrations of FAM-labelled aptamer (left to right -10 nM, 50 nM, 100 nM, 250 nM) JN27 (red) and JN27.SH (blue) were incubated with *P*. *aeruginosa* PA692. Cells were washed, re-suspended in buffer and analysed by flow cytometry to look for an increase in median fluorescence compared to bacteria with no aptamer (black).
(TIF)

S4 Fig Analysis of aptamer effect on the growth of PA692. 1 μM of each aptamer was incubated with 10^4^ CFU of bacteria and the growth rate followed by OD_600_ measurement every 30 min for 16 hours. No change in growth kinetics was seen between aptamer and no aptamer.
(TIF)
