# Supplementary figures and images for: Selection of DNA aptamers specific for live Pseudomonas aeruginosa
Source: PLoS One. 2017 Sep 22;12(9):e0185385. doi: 10.1371/journal.pone.0185385 (PMC5609762; doi:10.1371/journal.pone.0185385)

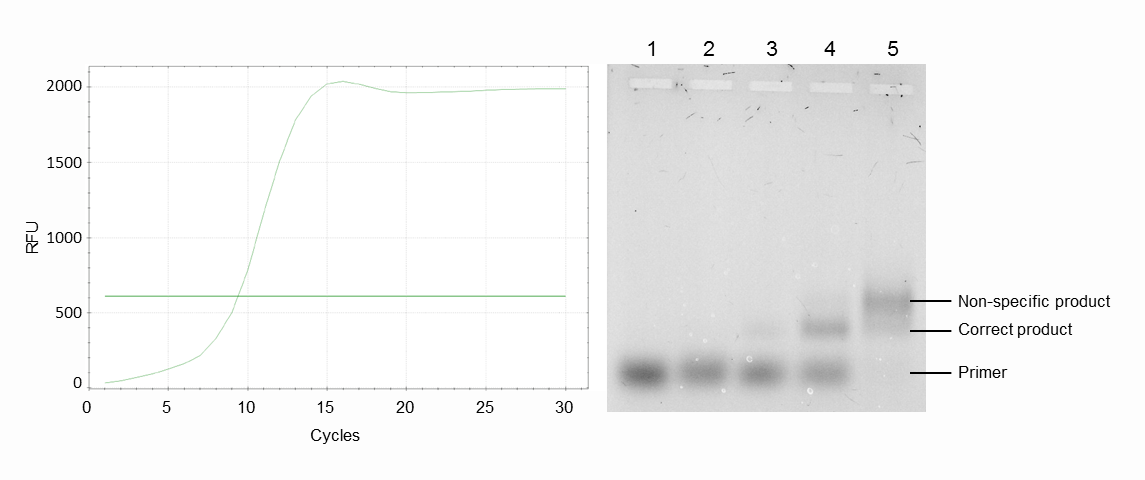

Supplement: S1 Fig — Aptamer PCR amplification can be monitored by real time PCR using SYBR green interchelation. Agarose gel electrophoresis was performed on samples after 7, 9, 11, 13 and 15 cycles of amplification (lanes 1 to 5 respectively). (TIF) [file pone.0185385.s001.tif]

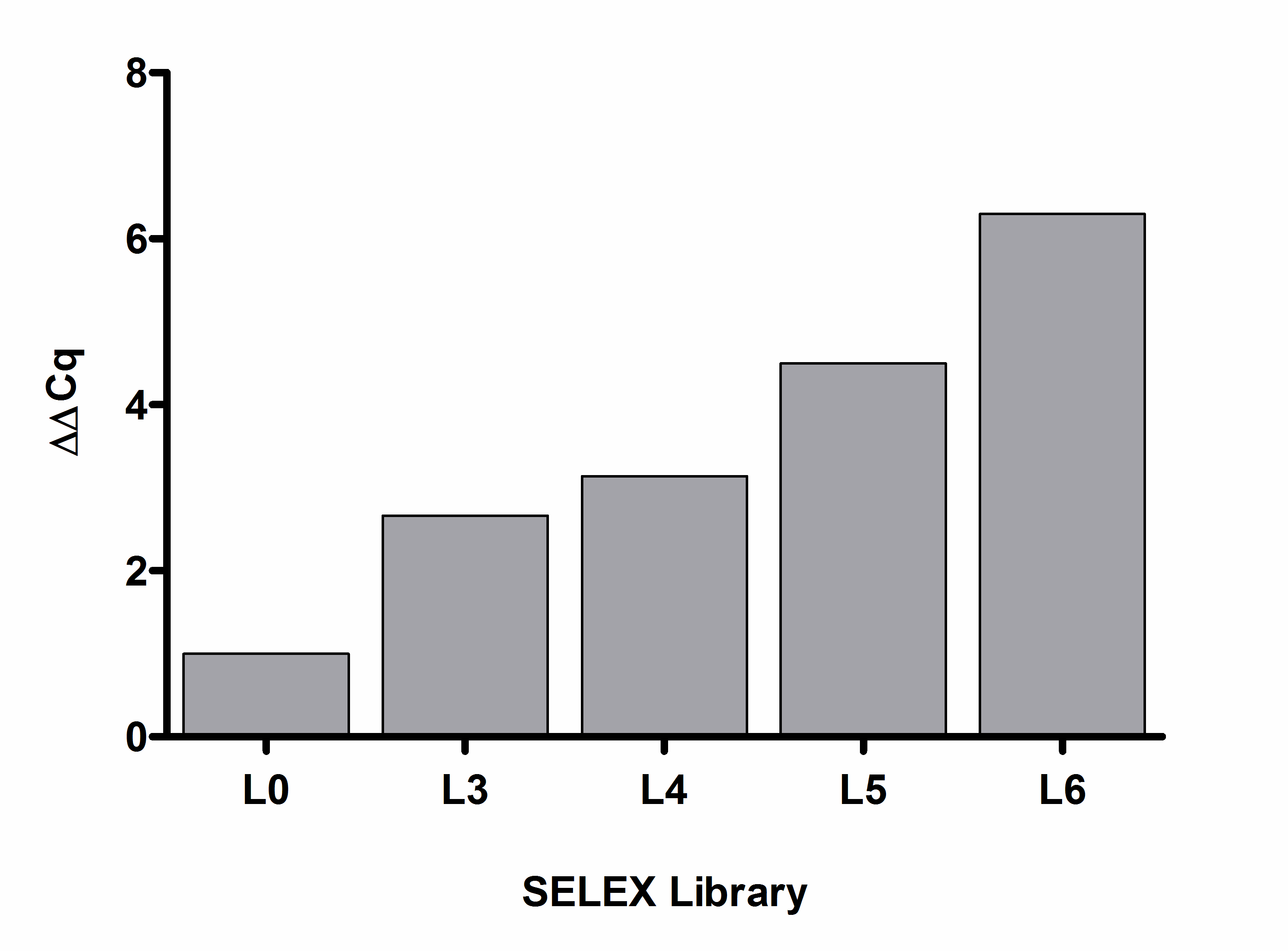

Supplement: S2 Fig — The relative binding of the aptamer libraries was determined by incubating 3x10^10^ biofilm bacterial cells collected from the flow cell with 100 nM of library in binding buffer for 30 min at room temperature. Samples were washed 3x, eluted and quantified by qPCR using the PCR protocol described in SELEX methods. The plot shows the change in the threshold cycle for amplification (Cq) relative to the initial starting library. (TIF) [file pone.0185385.s002.tif]

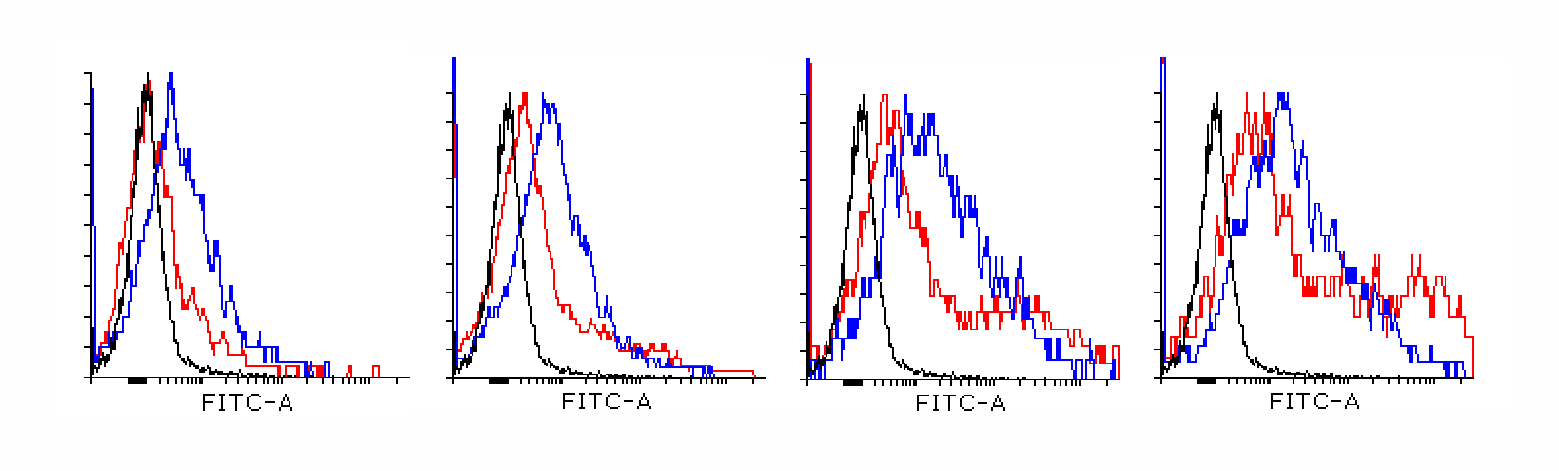

Supplement: S3 Fig — Increasing concentrations of FAM-labelled aptamer (left to right -10 nM, 50 nM, 100 nM, 250 nM) JN27 (red) and JN27.SH (blue) were incubated with P. aeruginosa PA692. Cells were washed, re-suspended in buffer and analysed by flow cytometry to look for an increase in median fluorescence compared to bacteria with no aptamer (black). (TIF) [file pone.0185385.s003.tif]

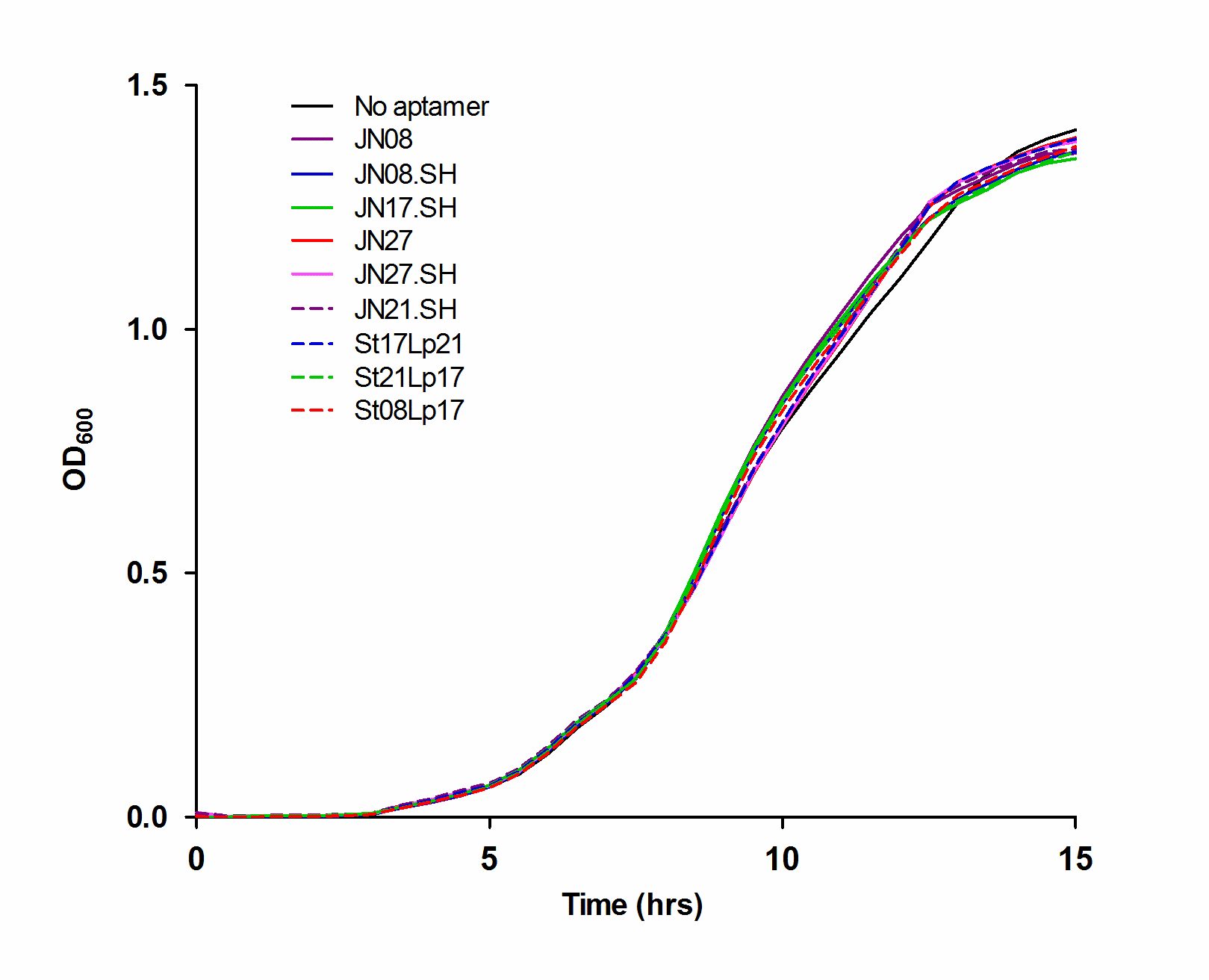

Supplement: S4 Fig — 1 μM of each aptamer was incubated with 104 CFU of bacteria and the growth rate followed by OD600 measurement every 30 min for 16 hours. No change in growth kinetics was seen between aptamer and no aptamer. (TIF) [file pone.0185385.s004.tif]
